# Patientensteuerung bei schweren psychischen Erkrankungen – Herausforderungen, Modelle und Perspektiven

**DOI:** 10.1007/s00103-026-04299-3

**Published:** 2026-09-16

**Authors:** Martin Lambert, Anja Zimmermann, Jürgen Gallinat

**Affiliations:** Klinik und Poliklinik für Psychiatrie und Psychotherapie, Zentrum für Psychosoziale Medizin, Universitätsklinikum Hamburg-Eppendorf (UKE), Martinistr. 52, 20246 Hamburg, Deutschland

**Keywords:** Patientensteuerung, Schwere psychische Erkrankungen, Assertive Community Treatment, Integrierte Versorgung, Stepped Care, Patient navigation, Severe mental illness, Assertive community treatment, Mental health care, Integrated care, Stepped care

## Abstract

Schwere psychische Erkrankungen („severe mental illness“, SMI) sind mit chronischen Verläufen, erheblichen funktionellen Einschränkungen und erhöhter Mortalität assoziiert. Gleichzeitig ist ihre Versorgung durch Fragmentierung, mangelnde Kontinuität und unzureichende Koordination gekennzeichnet. Vor diesem Hintergrund gewinnt Patientensteuerung als Ansatz zur Verbesserung von Versorgungsqualität und Effizienz an Bedeutung. Ziel dieser narrativen Übersichtsarbeit ist es, zentrale evidenzbasierte Versorgungsmodelle für Menschen mit SMI darzustellen, strukturelle Herausforderungen der Versorgung zu analysieren und daraus Prinzipien einer effektiven Patientensteuerung abzuleiten.

Die Evidenz zeigt, dass insbesondere kontinuierliche und multiprofessionelle Versorgungsansätze – wie Assertive Community Treatment (ACT; intensive gemeindenahe Behandlung), Crisis Resolution Teams (CRT; ambulante Krisenintervention durch multiprofessionelle Teams) und Frühinterventionsprogramme – klinische und funktionelle Outcomes verbessern, stationäre Behandlungen reduzieren und Kontinuität herstellen können. Gleichzeitig wird deutlich, dass die isolierte Implementierung einzelner Interventionsmodelle nicht ausreicht, um strukturelle Defizite nachhaltig zu adressieren.

Integrierte und gestufte Versorgungsmodelle ermöglichen die systematische Verknüpfung evidenzbasierter Interventionen entlang eines kontinuierlichen Versorgungspfades. Aktuelle Befunde zeigen, dass solche Ansätze die Versorgungsqualität verbessern und gleichzeitig ohne Mehrkosten implementiert werden können und sogar Einsparpotenziale erschließen. Patientensteuerung stellt damit einen zentralen Ansatz dar, um Versorgungskontinuität zu stärken, Versorgungsbrüche zu reduzieren und Patientenergebnisse sowie Ressourceneffizienz nachhaltig zu verbessern.

## Abstract (en)

Severe mental illness (SMI) is associated with chronic courses, significant functional impairments, and increased mortality. At the same time, care for this population is characterized by fragmentation, lack of continuity, and insufficient coordination. Against this background, patient navigation has gained importance as an approach to improving the quality and efficiency of care. The aim of this narrative review is to present key evidence-based models of care for individuals with SMI, to analyze structural challenges in service provision, and to derive principles for effective patient navigation.

The evidence shows that community-based, multidisciplinary, and continuous care approaches—such as assertive community treatment, crisis intervention, and early intervention programs—can improve clinical and functional outcomes, reduce hospitalizations, and enhance continuity of care. At the same time, it becomes clear that the isolated implementation of individual intervention models is insufficient to sustainably address structural deficits.

Integrated and stepped care models enable the systematic linkage of evidence-based interventions along a continuous care pathway. Current findings indicate that such approaches can improve quality of care while being implementable without additional costs and may even generate cost savings. Patient navigation thus represents a key strategy to strengthen continuity of care, reduce fragmentation, and sustainably improve patient outcomes as well as resource efficiency.

## Einleitung

Die Versorgung von Menschen mit schweren psychischen Erkrankungen (engl. „severe mental illness“, SMI) stellt eine zentrale Herausforderung für Gesundheitssysteme dar. SMI gehen häufig mit persistierenden Symptomen, erheblichen funktionellen Einschränkungen sowie hohen Raten psychischer und somatischer Komorbidität einher und sind mit einer um etwa 10–20 Jahre reduzierten Lebenserwartung assoziiert [1–6]. Insbesondere körperliche Multimorbidität trägt wesentlich zur erhöhten Mortalität und Krankheitslast bei und stellt eine zusätzliche Herausforderung für die Versorgung dar.

Die Versorgung von Menschen mit SMI ist in vielen Gesundheitssystemen strukturell defizitär. Charakteristisch sind eine fragmentierte Versorgungslandschaft, Defizite in der Implementierung evidenzbasierter Versorgungsmodelle sowie eine unzureichende sektorenübergreifende Koordination, die den Zugang zu leitliniengerechter Behandlung erschweren [1, 7–9]. Diese strukturellen Defizite begünstigen Chronifizierung, wiederholte stationäre Aufnahmen und Behandlungsabbrüche und führen in der Folge zu erheblichen individuellen und gesellschaftlichen Belastungen – sowohl für die Betroffenen als auch für ihre Bezugspersonen.

Vor diesem Hintergrund gewinnt der Begriff der Patientensteuerung zunehmend an Bedeutung. Darunter werden Ansätze verstanden, die den Zugang zur Versorgung sowie die Navigation innerhalb des Gesundheitssystems strukturieren und koordinieren, um eine bedarfsgerechte und effiziente Behandlung sicherzustellen. Patientensteuerung umfasst dabei sowohl unterstützende als auch direktive Elemente und bewegt sich im Spannungsfeld zwischen Versorgungsoptimierung und Patientenautonomie [10–12].

Für Menschen mit SMI ist eine effektive Patientensteuerung von besonderer Relevanz, da die Versorgung typischerweise mehrere Sektoren, Berufsgruppen und Institutionen umfasst und zugleich durch krankheitsbedingte Einschränkungen der Selbststeuerungsfähigkeit erschwert ist. Evidenzbasierte Versorgungsmodelle wie Frühinterventionsprogramme (Early Intervention Services, EIS), Assertive Community Treatment (ACT) und aufsuchende Kriseninterventionsansätze (Crisis Resolution Teams, CRT) zeigen positive Effekte, insbesondere hinsichtlich Versorgungskontinuität, Behandlungsbindung und der Reduktion stationärer Behandlungen; Effekte auf die Symptomreduktion sind hingegen weniger einheitlich [1, 13–23]. Dennoch ist ihre Implementierung in die deutsche Regelversorgung bislang begrenzt.

Ziel des vorliegenden Beitrags ist es, die Charakteristika schwerer psychischer Erkrankungen darzustellen, zentrale Versorgungsprobleme zu analysieren und evidenzbasierte Ansätze der Patientensteuerung einzuordnen. Darüber hinaus wird untersucht, wie Patientensteuerung für diese Hochrisikogruppe evidenzbasiert gestaltet werden sollte und wie sie aktuell in Deutschland umgesetzt ist.

## Definition und Konzept schwerer psychischer Erkrankung

Der Begriff der schweren psychischen Erkrankung („severe mental illness“, SMI) ist kein eigenständiger diagnostischer Terminus im Sinne von ICD-10/11 (Internationale statistische Klassifikation der Krankheiten und verwandter Gesundheitsprobleme, 10. und 11. Revision) oder DSM-5 (Diagnostisches und Statistisches Manual Psychischer Störungen, 5. Auflage), sondern ein versorgungsorientiertes Konzept zur Identifikation von Patientengruppen mit besonders hohem Behandlungs- und Unterstützungsbedarf [24–28]. Im Zentrum steht nicht die Diagnose allein, sondern die Kombination aus Schweregrad, Krankheitsverlauf und funktionellen Einschränkungen.

Schwere psychische Erkrankungen (SMI) werden typischerweise anhand von 4 zentralen Kriterien operationalisiert [24–28]:

1. das Vorliegen einer psychischen Störung,
2. eine ausgeprägte Symptomatik,
3. eine erhebliche und anhaltende Beeinträchtigung des psychosozialen Funktionsniveaus sowie
4. ein daraus resultierender erhöhter Unterstützungs- und Behandlungsbedarf.

Zur Abgrenzung persistierender Verläufe werden in der Literatur häufig Zeiträume von 6 bis 24 Monaten herangezogen, innerhalb derer eine anhaltende Einschränkung des psychosozialen Funktionsniveaus besteht [29]. Zur Erfassung dieser Einschränkungen kommen Instrumente wie das Global Assessment of Functioning (GAF) zum Einsatz; Werte ≤50 gelten als Hinweis auf eine relevante Beeinträchtigung, sollten jedoch aufgrund begrenzter Reliabilität nicht isoliert interpretiert werden [29].

Internationale Organisationen wie die Weltgesundheitsorganisation (WHO) beschreiben SMI als Störungen mit hoher Krankheitslast, ausgeprägter funktioneller Beeinträchtigung und erhöhtem Chronifizierungsrisiko [3, 30]. Zunehmend wird außerdem die Bedeutung körperlicher Multimorbidität betont, die wesentlich zur Krankheitslast und reduzierten Lebenserwartung beiträgt und damit integraler Bestandteil des SMI-Konzepts ist [3, 30].

Ein vergleichbares Verständnis findet sich in Deutschland in der KSVPsych-Richtlinie (Richtlinie über koordinierte und strukturierte Versorgung für schwer psychisch kranke Versicherte mit komplexem psychiatrischen oder psychotherapeutischen Behandlungsbedarf), die die Zielgruppe über einen komplexen Behandlungsbedarf unter Berücksichtigung von Diagnose, funktionellen Einschränkungen und multiprofessionellem Versorgungsbedarf definiert [31].

Konzeptionell werden SMI zunehmend als Kontinuum verstanden, entlang dessen sich unterschiedliche Verlaufs- und Schweregrade unterscheiden lassen. Hierzu zählen episodisch schwer verlaufende Störungen, anhaltende Verläufe mit persistierender Symptomatik sowie komplexe Krankheitsbilder mit multiprofessionellem und intersektoralem Versorgungsbedarf [26, 27, 29, 32]. Entsprechend können beispielsweise auch bestimmte Formen therapieresistenter Depressionen dem Spektrum der SMI zugeordnet werden.

Soziale Determinanten spielen eine wesentliche Rolle für Entstehung und Verlauf von SMI. Insbesondere Obdachlosigkeit steht in enger Wechselwirkung mit schweren psychischen Erkrankungen und ist sowohl Risikofaktor als auch Folge schwerer Krankheitsverläufe [33, 34]. Betroffene weisen häufig komplexe, chronische und multimorbide Verläufe auf. Versorgungsmodelle, die gesundheitliche und soziale Unterstützung integrieren, zeigen hier relevante Verbesserungen hinsichtlich Stabilisierung und Teilhabe [22, 35].

Die Verwendung des SMI-Konzepts ist insbesondere für Versorgungsplanung und -forschung relevant, da es die Identifikation einer Hochrisikogruppe mit erhöhtem Chronifizierungsrisiko, komplexem Unterstützungsbedarf und überproportionaler Inanspruchnahme von Gesundheitsleistungen ermöglicht.

### Einordnung für die Patientensteuerung

Das funktionale Verständnis von SMI impliziert, dass Versorgung nicht primär diagnosebasiert, sondern entlang von Schweregrad, Krankheitsverlauf und individuellem Bedarf organisiert werden sollte. Insbesondere bei persistierenden Verläufen ergibt sich ein Bedarf an langfristigen, intensiven und sektorenübergreifend koordinierten Versorgungsformen. Gleichzeitig ermöglicht das Kontinuumsmodell eine frühere Identifikation von Risikopatientinnen und -patienten und damit präventive Interventionen. Die Berücksichtigung sozialer Determinanten unterstreicht die Notwendigkeit niedrigschwelliger, aufsuchender und integrierter Steuerungsansätze.

## Epidemiologie schwerer psychischer Erkrankungen

Die Epidemiologie schwerer psychischer Erkrankungen ist aufgrund des versorgungsorientierten Charakters des Konzepts heterogen und abhängig von der jeweiligen Operationalisierung [24–28]. Entsprechend variieren Prävalenzschätzungen in Abhängigkeit von Diagnose, funktionellem Beeinträchtigungsgrad und Krankheitsdauer. Internationale Studien berichten 12-Monats-Prävalenzen im Bereich von etwa 1–2 %, während neuere Analysen – insbesondere aus den Vereinigten Staaten – Prävalenzraten von etwa 5–6 % zeigen [24, 26, 27]. Aktuelle Daten weisen zudem auf Unterschiede nach Geschlecht und Alter hin, mit höheren Raten bei Frauen sowie bei jüngeren Erwachsenen [26].

Das Risiko, eine schwere psychische Erkrankung zu entwickeln, variiert je nach zugrunde liegender Diagnose. Besonders hohe Risiken finden sich bei psychotischen Störungen, während auch bipolare Störungen und unipolare Depressionen in einem relevanten Anteil der Fälle schwere und persistierende Verläufe mit Funktionseinschränkungen annehmen können [26, 32]. Daher können in das Spektrum schwerer psychischer Erkrankungen auch therapieresistente Depressionen sowie schwere Persönlichkeits- und substanzbezogene Störungen eingehen.

Besonders hohe Prävalenzen finden sich in vulnerablen Bevölkerungsgruppen. Menschen in Obdachlosigkeit weisen deutlich erhöhte Raten psychischer Erkrankungen auf, wobei schwere psychische Erkrankungen bei bis zu 40–50 % der Betroffenen auftreten [34, 36, 37]. Diese sind häufig mit substanzbezogenen Störungen und somatischen Erkrankungen assoziiert und gehen mit ausgeprägter Multimorbidität einher.

Insgesamt zeigen die epidemiologischen Befunde, dass es sich bei SMI um eine vergleichsweise kleine, aber hoch belastete und versorgungsintensive Patientengruppe handelt. Gleichzeitig ist ein früher Erkrankungsbeginn häufig und bestimmte Bevölkerungsgruppen sind überproportional betroffen.

### Einordnung für die Patientensteuerung

Aus epidemiologischer Perspektive ergibt sich die Notwendigkeit risikoadaptierter und zielgruppenspezifischer Steuerungsansätze. Neben allgemeinen Zugangsstrukturen ist insbesondere die frühzeitige Identifikation von Hochrisikopatientinnen und -patienten von zentraler Bedeutung. Für vulnerable Gruppen – insbesondere Menschen in Obdachlosigkeit – sind niedrigschwellige, aufsuchende und integrierte Versorgungsansätze erforderlich, die gesundheitliche und soziale Unterstützung systematisch verbinden.

## Krankheitslast und Kosten

Schwere psychische Erkrankungen sind mit einer erheblichen individuellen, gesellschaftlichen und gesundheitsökonomischen Krankheitslast verbunden. Diese resultiert aus persistierenden Symptomen, funktionellen Einschränkungen, erhöhter Morbidität sowie einer hohen Inanspruchnahme von Gesundheits- und Sozialleistungen [1– 3, 6, 38–40].

### Individuelle Krankheitslast.

Auf individueller Ebene gehen SMI mit ausgeprägten Einschränkungen der Lebensqualität, sozialen Teilhabe und Erwerbsfähigkeit einher [1, 2, 41]. Die Beschäftigungsraten liegen häufig im Bereich von 20–30 % [6]. Gleichzeitig bestehen hohe Raten sozialer Isolation und Stigmatisierung. Ein zentraler Aspekt ist die deutlich reduzierte Lebenserwartung um etwa 10–20 Jahre, die nur teilweise auf Suizide, zu einem wesentlichen Teil jedoch auf somatische Komorbiditäten zurückzuführen ist [4–6, 42]. Diese tragen zudem zu ungünstigen Krankheitsverläufen, erhöhter Hospitalisierungsrate und geringerer Therapieadhärenz bei [3]. Die Krankheitsverläufe sind häufig durch Krisen, wiederholte stationäre Aufenthalte und Behandlungsabbrüche geprägt. Versorgungsbrüche stellen dabei einen wesentlichen Faktor für Chronifizierung und Verschlechterung dar [2, 8, 43].

### Krankheitslast in vulnerablen Gruppen.

Besonders ausgeprägt ist die Krankheitslast bei vulnerablen Bevölkerungsgruppen. Menschen in Obdachlosigkeit weisen häufig komplexe, chronische und multimorbide Krankheitsverläufe auf. SMI treten bei bis zu 40–50 % der Betroffenen auf und sind oft mit substanzbezogenen sowie somatischen Erkrankungen assoziiert [34, 36, 37]. Diese Population ist durch hohe Raten von Suizidalität, Multimorbidität und Mortalität gekennzeichnet sowie durch eine intensive, jedoch häufig ineffiziente Inanspruchnahme von Notfall- und stationären Leistungen („Drehtür“-Verläufe; [33, 39]).

### Gesellschaftliche und ökonomische Krankheitslast.

SMI verursachen erhebliche direkte und indirekte Kosten. Direkte Kosten entstehen vor allem durch stationäre und ambulante Behandlung sowie medikamentöse Therapie. Charakteristisch ist eine ausgeprägte Kostenkonzentration: Ein relativ kleiner Anteil der Patientinnen und Patienten verursacht einen großen Teil der Versorgungskosten [7, 44]. Indirekte Kosten infolge von Arbeitslosigkeit, Produktivitätsverlusten und Frühverrentung übersteigen häufig die direkten Kosten und können bis zu 2 Drittel der Gesamtkosten ausmachen [7].

### Inanspruchnahme und Kostenstruktur.

Die Inanspruchnahme von Gesundheitsleistungen ist bei SMI deutlich erhöht, insbesondere im stationären Bereich. Ein Großteil der direkten psychiatrischen Behandlungskosten entfällt auf stationäre Leistungen [7, 40, 45]. Gleichzeitig zeigen sich ineffiziente Nutzungsmuster mit wiederholten Kriseninterventionen, Notfallkontakten und vermeidbaren Hospitalisierungen, die Ausdruck mangelnder Versorgungskontinuität sind [7, 9]. Evidenzbasierte Versorgungsmodelle wie Frühinterventionsprogramme, Kriseninterventionsteams sowie aufsuchende und langfristig angelegte Behandlungsansätze zeigen, dass eine strukturierte und koordinierte Versorgung Hospitalisierungen reduzieren, die Versorgungskontinuität verbessern und zur Reduktion von Kosten beitragen kann [13, 16–18, 20, 43].

### Implikationen für die Patientensteuerung

Die Befunde zeigen eine starke Konzentration von Krankheitslast und Kosten auf eine relativ kleine Patientengruppe mit komplexen und chronischen Verläufen. Daraus ergibt sich die Notwendigkeit, Versorgungskontinuität zu sichern, Versorgungsbrüche zu vermeiden und ineffiziente Nutzungsmuster zu reduzieren. Zentrale Ansatzpunkte sind die frühzeitige Identifikation von Risikopatientinnen und -patienten, eine kontinuierliche und proaktive Versorgung sowie die Integration psychiatrischer, somatischer und sozialer Unterstützungsangebote.

## Versorgungsprobleme und strukturelle Herausforderungen

Trotz vorhandener evidenzbasierter Versorgungsmodelle ist die Versorgung von Menschen mit schweren psychischen Erkrankungen in Deutschland weiterhin durch strukturelle Defizite geprägt. Diese betreffen insbesondere den Zugang zur Versorgung, die Kontinuität von Behandlungsprozessen sowie die sektorenübergreifende Koordination [1, 2, 7–9].

### Zugang zur Versorgung.

Der Zugang zu leitliniengerechter Behandlung ist für viele Betroffene eingeschränkt. Ursachen sind unter anderem eine unzureichende Implementierung evidenzbasierter Versorgungsmodelle, begrenzte ambulante Kapazitäten, lange Wartezeiten sowie regionale Unterschiede in der Versorgungsdichte [2, 16]. Hinzu kommen krankheitsbedingte und soziale Faktoren wie eingeschränkte Krankheitseinsicht, Stigmatisierung und soziale Desintegration. Dadurch wird Versorgung häufig erst in fortgeschrittenen Krankheitsstadien oder akuten Krisensituationen in Anspruch genommen [1, 2, 9].

### Fragmentierung und mangelnde Koordination.

Die Versorgung ist häufig durch eine Trennung zwischen ambulanten, stationären und komplementären Angeboten gekennzeichnet. Diese Fragmentierung erschwert eine kontinuierliche Behandlung und führt zu Schnittstellenproblemen, insbesondere bei Übergängen zwischen Versorgungssettings [8, 43]. Zudem besteht eine unzureichende Integration somatischer und psychiatrischer Versorgung, was angesichts der hohen somatischen Multimorbidität die Versorgungsqualität zusätzlich beeinträchtigt [3, 6].

### Mangelnde Versorgungskontinuität.

Viele Patientinnen und Patienten mit SMI wechseln wiederholt zwischen verschiedenen Behandlungssettings, wobei die Versorgung häufig durch Phasen unzureichender Versorgung unterbrochen wird. Diese Diskontinuität erhöht das Risiko für Krankheitsverschlechterungen, Kriseninterventionen und Wiederaufnahmen [8, 43]. Besonders kritisch sind Übergänge nach stationären Aufenthalten, bei denen eine fehlende Anschlussversorgung ein wesentlicher Risikofaktor für erneute Hospitalisierungen ist [43].

### Ineffiziente Inanspruchnahmemuster.

Die beschriebenen strukturellen Defizite führen häufig zu einer reaktiven, krisenzentrierten Versorgung mit wiederholten Notfallkontakten, kurzfristigen stationären Aufenthalten und sogenannten Drehtür-Verläufen [7, 9]. Diese Nutzungsmuster sind klinisch ungünstig und mit erheblichen Kosten verbunden [40].

### Besondere Herausforderungen bei vulnerablen Gruppen.

Die Versorgungsprobleme sind bei vulnerablen Bevölkerungsgruppen besonders ausgeprägt. Menschen in Obdachlosigkeit weisen zusätzliche Zugangshürden auf, darunter instabile Lebensverhältnisse, geringe Anbindung an reguläre Versorgungsstrukturen sowie hohe Raten psychischer und somatischer Komorbidität. Ihre Versorgung erfolgt häufig überwiegend über Notfall- und Akutangebote, während kontinuierliche und präventive Leistungen unzureichend genutzt werden [33, 39].

### Systemische Rahmenbedingungen.

Auf Systemebene erschweren fragmentierte Finanzierungsstrukturen, sektorale Zuständigkeiten und fehlende Anreize für koordinierte Versorgung eine effektive Steuerung [7]. Die Versorgung ist häufig angebotsorientiert und reagiert primär auf akute Bedarfe, anstatt proaktiv und kontinuierlich zu intervenieren.

### Einordnung für die Patientensteuerung

Die bestehenden Versorgungsstrukturen sind häufig nicht geeignet, die komplexen und langfristigen Bedarfe von Menschen mit SMI adäquat abzubilden. Fragmentierung, mangelnde Kontinuität und ineffiziente Inanspruchnahme stellen zentrale Ansatzpunkte für Verbesserungen dar. Patientensteuerung zielt daher auf die Organisation kontinuierlicher, bedarfsadaptierter Versorgungspfade, die Versorgungsbrüche reduzieren, Übergänge strukturieren und eine frühzeitige sowie proaktive Betreuung ermöglichen. Vor allem für besonders vulnerable Gruppen sind niedrigschwellige, aufsuchende und intersektoral integrierte Ansätze erforderlich.

## Evidenzbasierte Versorgungsmodelle

Evidenzbasierte Versorgungsmodelle für Menschen mit schweren psychischen Erkrankungen adressieren zentrale strukturelle Defizite wie eingeschränkten Zugang zu leitliniengerechter Behandlung, Fragmentierung, mangelnde Kontinuität und unzureichende Koordination. Studien zeigen, dass insbesondere gemeindenahe, multiprofessionelle und kontinuierliche Versorgungsansätze klinisch relevante Verbesserungen erzielen, vor allem hinsichtlich Versorgungskontinuität, Behandlungsbindung und Reduktion stationärer Inanspruchnahme.

### Gemeindenahe und integrierte Versorgungsansätze.

Assertive Community Treatment (ACT) und Intensive Case Management (ICM) gehören zu den am besten untersuchten Versorgungsmodellen für Menschen mit SMI. Charakteristisch sind multiprofessionelle Teams, eine aufsuchende Betreuung sowie eine langfristige Begleitung. Metaanalysen zeigen konsistent eine Reduktion stationärer Aufenthalte, eine verbesserte Versorgungskontinuität und eine höhere Behandlungsbindung; Effekte auf die Symptomreduktion sind weniger einheitlich [18, 19, 23, 45]. Weiterentwicklungen dieser Ansätze integrieren ACT zunehmend in bestehende Versorgungssysteme und kombinieren es mit strukturierten therapeutischen Interventionen. Das Hamburger Modell (ACCESS) stellt ein Beispiel für eine solche Integration dar und zeigt Verbesserungen klinischer und funktioneller Outcomes sowie eine Reduktion von Hospitalisierungen und Zwangsmaßnahmen [46–51].

### Krisenintervention und Übergangsversorgung.

Kriseninterventionsmodelle wie CRT ermöglichen eine intensive stationsersetzende Behandlung im häuslichen Umfeld und tragen zur Reduktion stationärer Aufenthalte bei [20, 21]. In Deutschland stellt die stationsäquivalente Behandlung (StäB) einen vergleichbaren Ansatz dar [52]. Übergangsinterventionen, insbesondere nach stationären Aufenthalten, sind ein zentraler Baustein zur Sicherung von Versorgungskontinuität. Sie reduzieren Wiederaufnahmen und stabilisieren Behandlungsverläufe [43].

### Frühintervention und zielgruppenspezifische Ansätze.

Frühinterventionsprogramme bei psychotischen Erstmanifestationen verbessern klinische und funktionelle Outcomes und reduzieren langfristig chronische Verläufe [13]. Für besonders vulnerable Gruppen, insbesondere Menschen in Obdachlosigkeit, stellt ACT in Kombination mit sozialintegrativen Ansätzen eine wirksame Versorgungsstrategie dar. Studien zeigen Verbesserungen der Wohnstabilität, eine bessere Anbindung an Versorgungsstrukturen sowie eine Reduktion von Notfallkontakten und Hospitalisierungen [22].

### Modellvorhaben nach § 64b SGB V und KSVPsych-Richtlinie.

Ein zentraler, im deutschen Versorgungskontext besonders relevanter Ansatz sind Modellvorhaben nach § 64b SGB V. Diese seit 2012 implementierten Modelle zielen auf eine sektorenübergreifende, flexible und bedarfsadaptierte Versorgung schwer psychisch erkrankter Menschen ab. Charakteristisch ist die Überwindung starrer sektoraler Grenzen durch die Einführung eines Gesamtbudgets, das eine flexible Steuerung zwischen stationären, teilstationären und ambulanten Behandlungsformen ermöglicht. Ziel ist es, die Versorgung stärker am individuellen Bedarf auszurichten und stationäre Aufenthalte zu reduzieren [53, 54].

Empirische Evaluationen zeigen, dass Modellvorhaben nach § 64b SGB V [53, 54]:

- die Versorgungskontinuität verbessern,
- die Inanspruchnahme stationärer Leistungen reduzieren,
- eine stärkere ambulante und aufsuchende Versorgung ermöglichen und
- ohne zusätzliche Kosten umgesetzt werden können.

Neuere Evaluationen und Metaanalysen bestätigen diese Befunde und weisen zusätzlich auf Verbesserungen klinischer und funktioneller Outcomes sowie auf eine stärkere Ambulantisierung und Flexibilisierung der Versorgung hin [55–57]. Zentrale Elemente wie gestufte Versorgung, multiprofessionelle Teams sowie aufsuchende Ansätze weisen dabei deutliche Parallelen zu internationalen Modellen wie ACT und CRT auf.

Ergänzend wurde mit der KSVPsych-Richtlinie ein weiterer strukturierter Ansatz zur Verbesserung der Versorgung von Menschen mit SMI in die Regelversorgung eingeführt [31]. Die KSVPsych-RL verfolgt das Ziel, durch eine koordinierte, multiprofessionelle und sektorenübergreifende Versorgung die Behandlungsqualität und Kontinuität zu verbessern. Zentrale Elemente sind:

- die strukturierte Koordination der Versorgung,
- die Einbindung multiprofessioneller Behandlungsteams sowie
- die Förderung sektorenübergreifender Zusammenarbeit.

Erste Analysen und konzeptionelle Bewertungen weisen darauf hin, dass die KSVPsych-RL insbesondere das Potenzial hat, bestehende Versorgungsstrukturen stärker zu vernetzen und die Koordination komplexer Behandlungsverläufe zu verbessern [58]. Sowohl Modellvorhaben nach § 64b SGB V als auch die KSVPsych-RL stellen damit praxisrelevante Beispiele für die Umsetzung evidenzbasierter Patientensteuerung im deutschen Versorgungssystem dar und verdeutlichen die Bedeutung strukturierter, koordinierter und bedarfsadaptierter Versorgungsansätze.

### Digitale und hybride Versorgungsansätze.

Digitale Interventionen, einschließlich telemedizinischer Angebote und Monitoring-Systeme, können Versorgungskontinuität und Adhärenz unterstützen. Erste Studien zeigen positive Effekte, insbesondere in Kombination mit bestehenden Versorgungsstrukturen, die Evidenz ist jedoch noch heterogen [59].

### Integrierte und gestufte Versorgungsmodelle als Gesamtlösung.

Vor dem Hintergrund der beschriebenen Einzelansätze gewinnen integrierte und gestufte Versorgungsmodelle zunehmend an Bedeutung. Diese kombinieren evidenzbasierte Interventionen entlang eines kontinuierlichen Versorgungspfades und passen die Behandlungsintensität dynamisch an den individuellen Bedarf an. Ein Beispiel hierfür ist das RECOVER-Modell, das gemeindenahe Behandlung, Krisenintervention, strukturierte Diagnostik und koordinierte Langzeitbetreuung in einem integrierten Versorgungssystem zusammenführt. Studien zeigen, dass solche Modelle sowohl klinische und funktionelle Outcomes verbessern als auch die Versorgung effizienter gestalten können [16, 17].

### Einordnung für die Patientensteuerung

Die dargestellten Modelle verdeutlichen, dass evidenzbasierte Interventionen insbesondere dann wirksam sind, wenn sie nicht isoliert, sondern als Bestandteil integrierter Versorgungssysteme implementiert werden. Modelle wie ACT, CRT, § 64b SGB V und integrierte Versorgungssysteme zeigen, dass eine strukturierte, koordinierte und bedarfsadaptierte Patientensteuerung sowohl klinische als auch ökonomische Vorteile bietet. Sie bilden damit die konzeptionelle Grundlage für eine effektive Patientensteuerung bei SMI.

## Prinzipien einer evidenzbasierten Patientensteuerung

Die vorliegenden Befunde verdeutlichen, dass die Versorgung von Menschen mit SMI eine strukturierte und sektorenübergreifende Patientensteuerung erfordert. Ziel ist die Organisation kontinuierlicher, bedarfsadaptierter Versorgungspfade, die Versorgungsbrüche reduzieren und eine effiziente sowie leitliniengerechte Behandlung ermöglichen.

*Strukturierter Zugang zu evidenzbasierter Versorgung:* Ein zentrales Prinzip sind die Früherkennung sowie der frühzeitige und systematische Zugang zu evidenzbasierten Versorgungsmodellen. Frühinterventionsprogramme (EIS), Kriseninterventionsmodelle (CRT) und Assertive Community Treatment (ACT) verbessern Versorgungskontinuität und reduzieren stationäre Behandlungen. Für die Patientensteuerung ergibt sich die Notwendigkeit, diese Angebote regional verfügbar zu machen und den Zugang insbesondere für Hochrisikopatientinnen und -patienten aktiv zu organisieren.

*Kontinuität und klare Versorgungsverantwortung:* Kontinuierliche Betreuung über verschiedene Krankheitsphasen hinweg ist entscheidend für stabile Verläufe. Hierfür sind klar definierte Zuständigkeiten notwendig, die auch sektorenübergreifend bestehen bleiben. Modelle wie integrierte Versorgungssysteme oder § 64b SGB V zeigen, dass eine solche kontinuierliche Versorgungsverantwortung im deutschen System grundsätzlich realisierbar ist.

*Proaktive und aufsuchende Versorgung:* Eine rein reaktive Versorgung ist bei SMI häufig unzureichend. Proaktive und aufsuchende Ansätze ermöglichen eine frühzeitige Intervention, verbessern die Behandlungsbindung und reduzieren Krisen sowie stationäre Aufnahmen. Dies umfasst insbesondere regelmäßige Kontaktangebote, aktive Nachverfolgung und aufsuchende Betreuung bei besonders gefährdeten Patientengruppen.

*Sektorenübergreifende Koordination und Entlassmanagement:* Die strukturierte Verknüpfung ambulanter, stationärer und komplementärer Versorgungsangebote ist zentral für eine effektive Patientensteuerung. Übergänge zwischen Versorgungssettings stellen dabei kritische Punkte dar. Ein strukturiertes Entlassmanagement mit frühzeitiger Planung der Anschlussversorgung, klaren Ansprechpartnern und verbindlicher Kommunikation ist essenziell, um Versorgungslücken und Wiederaufnahmen zu vermeiden.

*Bedarfsadaptierte und gestufte Versorgung:* Die Behandlungsintensität sollte dynamisch an den individuellen Bedarf angepasst werden. Integrierte und gestufte Versorgungsmodelle ermöglichen es, evidenzbasierte Interventionen entlang eines kontinuierlichen Versorgungspfades flexibel zu kombinieren und Über- sowie Unterversorgung zu vermeiden.

*Integration somatischer und sozialer Versorgung:* Aufgrund der hohen somatischen Komorbidität und sozialer Belastungen ist eine integrierte Versorgung erforderlich. Die koordinierte Einbindung medizinischer, psychosozialer und sozialer Unterstützungsangebote ist entscheidend für eine nachhaltige Stabilisierung und Verbesserung der Lebensqualität.

*Niedrigschwelliger Zugang und Zielgruppenorientierung:* Versorgungsstrukturen müssen niedrigschwellig gestaltet und gezielt auf schwer erreichbare Gruppen ausgerichtet werden. Insbesondere bei Menschen in Obdachlosigkeit sind aufsuchende und integrierte Ansätze notwendig, um bestehende Versorgungslücken zu schließen.

*Digitale Unterstützung der Patientensteuerung:* Digitale Technologien können Patientensteuerung insbesondere durch verbessertes Monitoring, Kommunikation und Koordination unterstützen. Sie ermöglichen eine höhere Transparenz von Behandlungsverläufen und können zur frühzeitigen Erkennung von Krisen beitragen.

## Fazit

Für die Weiterentwicklung der Patientensteuerung im deutschen Versorgungssystem ergibt sich die Notwendigkeit, bestehende Strukturen stärker zu vernetzen und evidenzbasierte Modelle systematisch zu implementieren. Ansätze wie § 64b SGB V und die KSVPsych-Richtlinie zeigen, dass sektorenübergreifende und koordinierte Versorgung grundsätzlich umsetzbar ist, ihre flächendeckende Implementierung steht jedoch noch aus.

Zentrale Entwicklungsschritte sind:

- die Früherkennung schwerer psychischer Erkrankungen;
- der Ausbau evidenzbasierter, integrierter und sektorenübergreifender Versorgungsstrukturen;
- die verbindliche Etablierung von Koordinationsfunktionen;
- die systematische Gestaltung von Übergängen zwischen Versorgungssettings sowie
- die stärkere Nutzung digitaler Unterstützungssysteme.

Effektive Patientensteuerung basiert auf Kontinuität, Proaktivität, Koordination und Bedarfsadaptation. Ziel ist die Etablierung durchgängiger, sektorenübergreifender Versorgungspfade, die Versorgungsqualität, Patientenergebnisse und Ressourceneffizienz nachhaltig verbessern.

## Data Availability

Alle dieser Arbeit zugrunde liegenden Daten sind in diesem Artikel enthalten.
